# Spoon-handle Obstetric Forceps

**Published:** 1888-07

**Authors:** Jno. Stainback Wilson

**Affiliations:** Atlanta, Ga.


### THE SPOON-HANDLE OBSTETRIC FORCEPS.

BY JNO. STAINBACK WILSON, M.D., ATLANTA, GA.

Reading in the "Transactions of the Medical Association of Georgia, of 1880," the highly interesting case of concentrated pelvis complicated with puerperal convulsions, in which a spoon-handle was used as a lever by the reporter, Dr. G. F. Worsen, I am reminded of a similar case in my own practice, without however entering any claim to priority. The title above may appear somewhat strange, even in this day when we have such an endless number of pessaries, forceps, and other gynecological instruments with unpronouncable French, German and Russian names. In this grand array of inventions, why should we not have the plain, *spoon-handle*, *American* forceps? So far as it has been tested it has proved to be a very safe and efficient instrument, and it has the advantage of being cheap, readily obtainable, and then we have no trouble in calling or remembering the name.

But to the case. After the close of the late war, I found myself engaged in a country practice, in Texas, with no superabundance of surgical instruments, or indeed, anything else. I had inherited from my father a pair of obstetric forceps which were long, double-curved, thick, clumsy, and apparently better adapted to deliver an elephant than a woman; and even these were out of place and unattainable at the time. Thus unequipped, I was called to a stout young woman in labor with her first child. She was of full habit, in fair health, but her skin and eyes indicated malarial impregnation, which was quite common in that region, (the Lower Cross Timbers of Middle Texas) where every form of periodical fevers of the most malignant type prevailed. I merely mention this incidentally, because I may call attention to a fact of which I am firmly convinced, and that is, that malarial poisoning is a frequent cause of puerperal convulsi ns.

Everything went on well both as to the duration of the labor and the character of the pains until the head of the child began to press strongly on the somewhat rigid perienum, when without any premonition she was seized with a convulsion.

Some idea of the violence of the convulsions, which recurred in rapid succession, may be formed when I states truthfully that the house could be felt to quiver and shake with each return of the clonic spasms. But lest I may be charged with exaggeration, it is proper to say that houses in that part of Texas are built mostly by nailing thick planks twelve or fourteen inches wide upright, to a frame of scantling. With the convulsions, the pains ceased almost entirely, and everything come to a *stand still*, except the terrible convulsions, which come on in rapid succession, with the shocking accompaniments of bloody froth from the mouth, distorted eyeballs, stertorous breathing, coma, etc.

I bled her as soon as the first paroxysm passed off, and administered chloroform by inhalation; but the convulsions continuing, and the pains being ineffective, I knew that the only certain and permanent relief was delivery as soon as possible.

Some fourteen miles distant lived a village phvsician, who, I presumed, must be provided with forceps, practicing as he did in a place of some size, in this age of progress when such an instrument is considered as essential to a physician's outfit as his pocket case or his gum lancet. During the interval of three or four hours until the arrival of my assistant, I managed to keep the convulsions somewhat under control by the free use of chloroform; but the pains grew weaker, and the head of the child made not a particle of progress.

After so long a time—how long those who have been placed in similar circumstances, can form some conception—the consulting, rather the much-desired forceps-bearing physician arrived, but without the forceps, he being in th same condition as myself as to the non-possession of this sometimes indispensable instrument. In this case, there was no disagreement as to what was necessary to be done, and the doctors did not differ, as they are often charged with doing; but *how?* We could not so readily decide. Ergot was too slow and uncertain, if not positively contraindicated by her exhausted condition. Instrumental delivery was indicated beyond all question, but no instruments could be had, unless we could extemporize something about the place. Necessity being the mother of invention, it was agreed that we get two tablespoons from the kitchen, and make a pair of forceps from these, or at any rate something that would do the work of forceps. These spoons were made of pewter or some composition metal, and could be bent without much difficulty. The handles were bent at an obtuse angle—about 45° degrees—two or three inches from the end. The two handles were hooked on each side of the occipital protuberance, my assistant holding the bowl part of one spoon and I the other. Then each approximating his hand to the side of the head, we made pressure sufficient to prevent slipping, and then drawing in concert, simultaneous with a slight pain, our suffering patient was delivered the first effort.

No comment is needed. The practical lesson is that much may be done by simple means, and that country doctors may, by the exercise of their inventive faculties, get along well without all the armamenta of modern surgery and gynecology.

It may be objected that the spoons used in the manner indicated did not act as forceps, but as a lever. But as a rose by any other name would smell as sweet, we will not dispute as to the name. Call the instrument lever or forceps, it answered all the purposes of the most complicated and costly instruments.

